# Supplementary material for: Week 96 Results of Switching from Tenofovir Disoproxil Fumarate-Based Antiretroviral Therapy to Coformulated Elvitegravir, Cobicistat, Emtricitabine, and Tenofovir Alafenamide among HIV/Hepatitis B Virus-Coinfected Patients
Source: Microbiol Spectr. 2023 Mar 29;11(3):e05125-22. doi: 10.1128/spectrum.05125-22 (PMC10269761; doi:10.1128/spectrum.05125-22)

Supplementary Materials

Supplementary Table 1. Demographic and clinical characteristics of the participants at baseline and weeks 48, 72, and 96

	Baseline n=274	Week 48 n=261	Week 72 n=244	Week 96 n=230	Baseline vs. Week 96, *p*
Age, median (IQR), years	41 (36-47)	42 (37-48)	43 (38-49)	43 (38-49)	
Male sex, n (%)	269 (98.2)	257 (98.5)	243 (99.6)	230 (100)	
Men who have sex with men, n (%)	238 (86.9)	229 (87.7)	219 (89.8)	208 (90.4)	
Injection drug users, n (%)	20 (7.3)	12 (4.6)	12 (4.9)	10 (4.3)	
Years since HIV diagnosis, median (IQR)	7.3 (4.0-10.8)	8.2 (5.1-11.7)	8.5 (5.5-12.2)	9.0 (6.1-12.7)	
Duration of TDF use, median (IQR), years	4.0 (2.4-6.0)	5.1 (3.4-7.0)	5.6 (4.0-7.5)	6.2 (4.5-8.0)	
Weight[†], median (IQR), kg	69 (60-77)	72 (64-79)	NA	72 (65-79)	<0.001
Anti-HCV positivity, n (%)	36/266 (13.5)	27/170 (15.9)	NA	18/200 (9.0)	0.375
Anti-HDV positivity, n (%)	40 (14.6)	37/260 (14.2)	30/241 (12.4)	30/224 (13.4)	0.375
Positive RPR titer, n (%)	119/271 (43.9)	112/251 (44.6)	114/228 (50.0)	97/218 (44.5)	0.230
Plasma HIV RNA <50 copies/mL, n (%)	274 (100)	258/260 (99.2)	238/244 (97.5)	216/230 (93.9)	<0.001
CD4 count, median (IQR), cells/μL	570 (433-721)	588 (439-742)	595 (440-773)	634 (456-779)	<0.001
ALT, median (IQR), U/L	26 (20-37)	24 (17-33)	23 (17-35)	24 (18-36)	0.092
AST, median (IQR), U/L	25 (21-31)	22 (19-29)	22 (19-27)	23 (19-31)	0.067
Cirrhosis of the liver, n (%)	4/250 (1.6)	4/170 (2.4)	NA	2/141 (1.4)	0.999
Serum creatinine, median (IQR), mg/dL	0.94 (0.84-1.08)	1.00 (0.90-1.10)	0.98 (0.88-1.08)	1.00 (0.90-1.10)	<0.001
eGFR, median (IQR), mL/min/1.73m^2	98.8 (85.6-109.2)	94.9 (82.4-105.5)	94.7 (83.2-106.3)	93.5 (82.7-102.7)	<0.001
HBV DNA and serological markers					
Plasma HBV DNA <20 IU/mL, n (%)	258 (94.2)	246/260 (94.6)	226/244 (92.6)	216/230 (93.9)	0.999

HBeAg positivity, n (%)	35 (12.8)	33/260 (12.7)	NA	23/226 (10.2)	
Anti-HBe positivity, n (%)	206 (75.2)	192/260 (73.8)	NA	172/226 (76.1)	
HBsAg positivity, n (%)	274 (100)	257/260 (98.4)	239/242 (98.8)	225/227 (99.1)	
HBsAg level, median (IQR), IU/mL	678 (90-1703)	656 (95-1590)	558 (82-1651)	566 (68-1495)	<0.001
HBsAg level, median (IQR), Log_{10} IU/mL	2.8 (2.0-3.2)	2.8 (2.0-3.2)	2.8 (1.9-3.2)	2.8 (1.8-3.2)	<0.001
Positive or equivocal anti-HBs Ab, n (%)	5 (1.8)	6/260 (2.3)	NA	3/226 (1.3)	
ALT normalization according to central laboratory (ALT ≤ 40 U/L)	NA	26/49 (53.1)	24/44 (54.5)	22/39 (56.4)	
ALT normalization according to 2018 AASLD criteria*	NA	37/72 (51.4)	32/66 (48.5)	30/60 (50)	

†Only 89 patients had body weight data at both baseline and week 48

*ALT ≤ 35U/L for males and ≤ 25 U/L for females

Abbreviations: AASLD, American Association for the Study of Liver Diseases; ALT, alanine transaminase*;* AST, aspartate transaminase*;* eGFR, estimated glomerular filtration rate; HBeAg, hepatitis B virus envelope antigen; HBsAg, hepatitis B surface antigen; HCV, hepatitis C virus; HDV, hepatitis D virus; IQR, interquartile range; NA, not applicable; NNRTI, non-nucleoside reverse-transcriptase inhibitor; RPR, rapid plasma reagin; TDF, tenofovir disoproxil fumarate

Supplementary Table 2. Changes in the renal function, urine protein, and metabolic profiles of the participants at baseline, weeks 48, 72, and 96

	Baseline n=274	Week 48 n=261	Week 72 n=244	Week 96 n=230	Baseline vs Week 96, p
**Renal function**					
Serum creatinine, median (IQR), mg/dL	0.94 (0.84-1.08)	1.00 (0.90-1.10)	0.98 (0.88-1.08)	1.00 (0.90-1.10)	<0.001
Estimated GFR, median (IQR), mL/min/1.73m^2	98.8 (85.6-109.2)	94.9 (82.4-105.5)	94.7 (83.2-106.3)	93.2 (82.3-102.6)	<0.001
Proteinuria on urine dipstick					
Absent	221 (81.0)	225 (86.5)	229 (95.8)	217 (96.0)	<0.001
Trace (15 to 30 mg/dL)	41 (15.0)	19 (7.3)	4 (1.7)	1 (0.4)	
1 + (30 to 100 mg/dL)	9 (3.3)	15 (5.8)	3 (1.3)	5 (2.2)	
≥ 2 + (100 to 300 mg/dL)	2 (0.7)	1 (0.4)	2 (1.2)	3 (1.3)	
UPCR, median (IQR), mg/g	79 (57-114)	68 (55-96)	68 (50-86)	63 (46-84)	<0.001
UPCR, median change from baseline (IQR), %	NA	-12.3 (-33.8-14.2)	-17.8 (-35.5-9.2)	-22.9 (-40.5-3.2	
UACR, median (IQR), mg/g	5.0 (3.1-9.8)	4.5 (3.0-8.3)	5.0 (6.2-9.2)	4.3 (2.9-7.5)	0.042
UACR, median change from baseline (IQR), %	NA	-15.3 (-46.4-50.0)	-3.2 (-35.6-82.9)	-12.5(-51.8-60.5)	
Urine β-2 microglobulin, median (IQR), ng/mL	228 (111-909)	128 (68-273)	115 (71-231)	120 (64-240)	<0.001
Urine β-2 microglobulin/Cr, median	165 (89-687)	90 (64-182)	93.5 (62.4-	83.0 (60.0-	<0.001

(IQR), μg/g			194.8)	170.1)	
Urine β-2 microglobulin/Cr, median change from baseline, median (IQR), %	NA	-38.5 (-70.6- -1.9)	-42.6 (-74.8- -5.5)	-51.3 (-77.1- -11.0)	
**Lipid profiles and blood glucose**					
Triglyceride median (IQR), mg/dL	116 (85-174)	140 (104-199)	139 (97-207)	141 (97-213)	<0.001
Triglyceride, median change from baseline (IQR), mg/dL	NA	26 (-17-73)	24 (-19-69)	27 (-8-72)	
Total cholesterol median (IQR), mg/dL	166 (149-193)	192 (167-220)	194 (166-226)	190 (170-222)	<0.001
Total cholesterol, median change from baseline (IQR), mg/dL	NA	23 (4-43)	30 (1-51)	25 (5-46)	
LDL-C, median (IQR), mg/dL	99 (84-118)	118 (97-137)	122 (95-144)	117 (95-137)	<0.001
LDL-C, median change from baseline (IQR), mg/dL	NA	19 (4-34)	20 (1-39)	17 (-1-35)	
HDL-C, median (IQR), mg/dL	42 (35-49)	46 (39-54)	48 (40-56)	47 (39-56)	<0.001
HDL-C, median change from baseline (IQR), mg/dL	NA	4 (-2-9)	5 (0-11)	5 (-1-11)	
T-CHO:HDL-C ratio, median (IQR)	4.0 (3.4-4.7)	4.2 (3.5-5.0)	4.1 (3.4-4.9)	4.1 (3.4-4.9)	<0.001
T-CHO/HDL-C ratio, median change from baseline (IQR)	NA	0.22 (-0.20-0.74)	0.20 (-0.32-0.73)	0.09 (-0.37-0.65)	
Fasting blood glucose, median (IQR), mg/dL	93 (86-101)	92 (87-100)	91 (85-98)	91 (85-100)	0.423
HbA1C, median (IQR), %	5.4 (5.2-5.7)	5.4 (5.1-5.6)	5.4 (5.2-5.7)	5.4 (5.1-5.7)	0.818

Abbreviations: Cr, creatinine; GFR, glomerular filtration rate; HDL-C, high-density lipoprotein cholesterol; IQR, interquartile range; LDL-C, low-density lipoprotein cholesterol; T-CHO, total cholesterol; UACR, urine albumin-to-urine creatinine ratio; UPCR, urine protein-to-urine creatinine ratio

Supplementary Table 3. Factors associated with declines of estimated glomerular filtration rate over 15% at week 96 by multivariate logistic regression

Variable	Univariate analysis		Multivariate analysis	
	Odds Ratio (95% confidence interval)	*p*	Odds Ratio (95% confidence interval)	*p*
Age, per 1-year increase	1.047 (1.001-1.094)	0.045		
Hypertension	1.907 (0.494-7.359)	0.349		
Diabetes	1.011 (0.118-8.697)	0.992		
CD4 count at baseline, per 1-cell/µL increase	0.998 (0.996-1.000)	0.038	0.997 (0.995-1.000)	0.019
Estimated GFR at baseline, per 1-mL/min/1.73m^2 increase	0.998 (0.975-1.021)	0.844		
Urine protein-to-creatinine ratio at baseline, per-1 mg/g increase	1.005 (1.002-1.008)	0.003	1.005 (1.001-1.008)	0.007
ART without creatinine transport inhibitors before switch	2.748 (1.008-7.494)	0.048	3.135 (1.013-9.697)	0.047

Abbreviations: ART, antiretroviral therapy; GFR, glomerular filtration rate

Supplementary Table 4. Changes in bone mineral density of the participants at baseline and weeks 48 and 96

		Baseline n=181	Week 48 n=159	Week 96 n=138	Baseline vs Week 96, p
Bone mineral density of the lumbar spine, median (IQR), g/cm^2		1.09 (1.01-1.21)	1.12 (1.03-1.23)	1.11 (1.02-1.20)	0.003
Lumbar spine T-score, median (IQR)		-0.3 (-1.0-0.6)	-0.1 (-0.8-0.8)	-0.1 (-0.7-0.7)	<0.001
Lumbar spine Z-score, median (IQR)		-0.1 (-0.8-0.8)	0.1 (-0.7-0.9)	0.0 (-0.6-0.8)	0.001
Lumbar spine	Normal, n (%)	140/180 (77.8)	128/158 (81.0)	114/137 (83.2)	
	Osteopenia, n (%)	37/180 (20.6)	29/158 (18.4)	21/137 (15.3)	
	Osteoporosis, n (%)	3/180 (1.7)	1/158 (0.6)	2/137 (1.5)	
Bone mineral density of the hip, median (IQR), g/cm^2		0.91 (0.81-1.00)	0.90 (0.82-1.02)	0.91 (0.82-1.03)	0.001
Hip T-score, median (IQR)		-0.5 (-1.2-0.3)	-0.5 (-1.1-0.4)	-0.3 (-1.1-0.5)	<0.001
Hip Z-score, median (IQR)		-0.2 (-0.9-0.5)	-0.1 (-0.8-0.7)	0.0 (-0.9-0.75)	<0.001
Hip	Normal, n (%)	125/181 (69.1)	118/159 (74.2)	101/137 (73.7)	
	Osteopenia, n (%)	50/181 (27.6)	39/159 (24.5)	32/137 (23.4)	
	Osteoporosis, n (%)	6/181 (3.3)	2/159 (1.3)	4/137 (2.9)	

Supplementary Table 5. Comparisons of clinical characteristics of participants who had positive anti-HDV IgG and those who had negative anti-HDV IgG at baseline

	Positive anti-HDV IgG n=40	Negative anti-HDV IgG n=234	*p*
Age, median (IQR), years	43 (38-47)	40 (36-47)	0.194
Male sex, n (%)	38 (95)	231 (98.7)	0.156
Men who have sex with men, n (%)	29 (72.5)	209 (89.3)	0.004
Injecting drug users, n (%)	13 (32.5)	7 (3.0)	<0.001
Years since HIV diagnosis, median (IQR)	9.9 (6.3-12.7)	6.9 (3.8-10.4)	0.005
Duration of TDF use, median (IQR), years	3.8 (2.4-5.9)	4.1 (2.4-6.0)	0.781
Anti-HCV positivity, n (%)	16/39 (41)	20/227 (8.8)	<0.001
Positive RPR titer, n (%)	23/40 (57.5)	96/231 (41.6)	0.061
CD4 count, median (IQR), cells/µL	651 (460-728)	557 (419-721)	0.127
ALT, median (IQR), U/L	26 (22-47)	26 (19-36)	0.143
AST, median (IQR), U/L	27 (23-42)	25 (21-30)	0.004
ALT >40 U/L, n (%)	12/40 (30)	42/234 (17.9)	0.077
Plasma HBV DNA <20 IU/mL, n (%)	39/40 (97.5)	219/234 (93.6)	0.330
HBeAg positivity, n (%)	4/40 (10)	31/234 (13.2)	0.570
Anti-HBe positivity, n (%)	28/40 (70)	178/234 (76.1)	0.412
HBsAg level, median (IQR), IU/mL	877 (105-1784)	674 (90-1663)	0.861
HBsAg level, median (IQR), Log_{10} IU/mL	2.9 (2.0-3.3)	2.8 (2.0-3.2)	0.876

Abbreviations: ALT, alanine transaminase*;* AST, aspartate transaminase*;* HBeAg, hepatitis B virus envelope antigen; HBsAg, hepatitis B surface antigen; HCV, hepatitis C virus; HDV, hepatitis D virus; IQR, interquartile range; RPR, rapid plasma reagin; TDF, tenofovir disoproxil fumarate

Supplementary Figure 1. Flow diagram of the study. E/c/F/TAF, coformulated elvitegravir, cobicistat, emtricitabine, and tenofovir alafenamide; BMD, bone mineral density; DDI, drug-drug interaction; TDF, tenofovir disoproxil fumarate

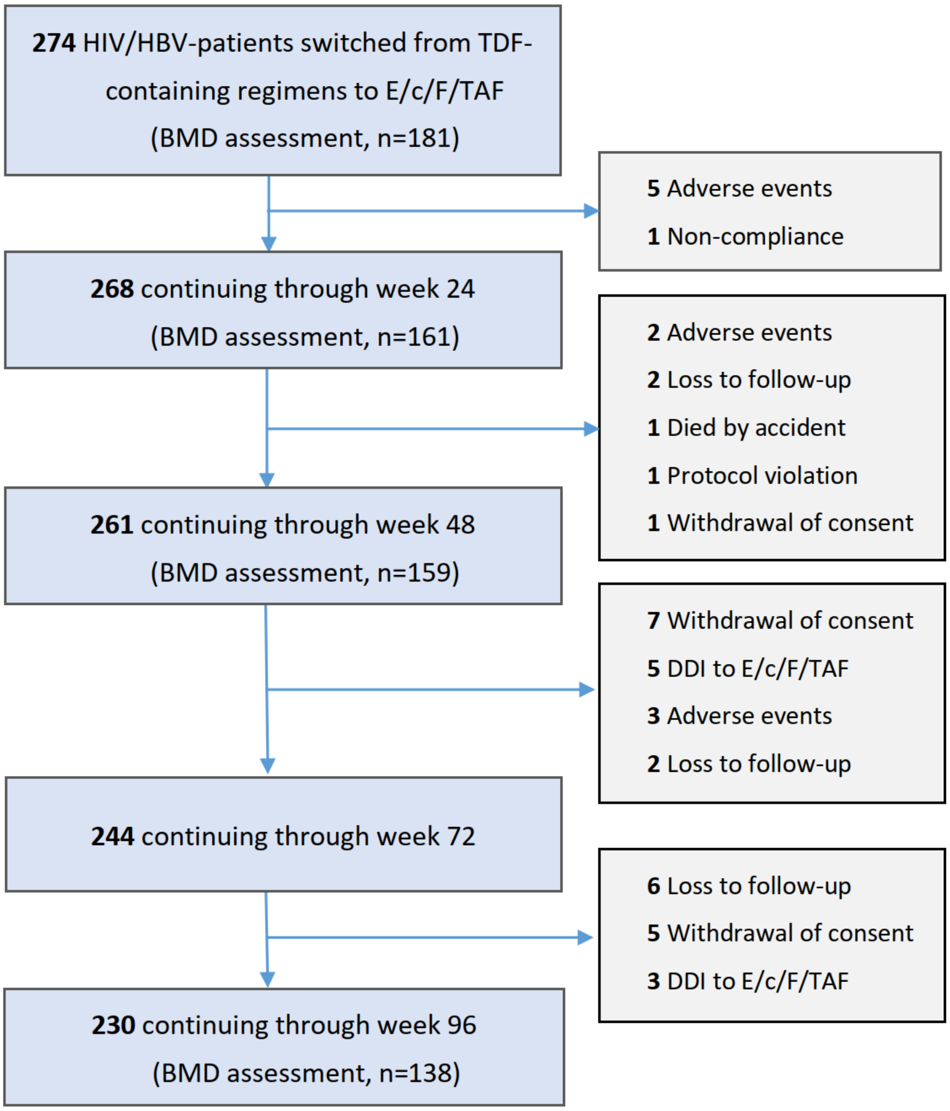

Supplementary Figure 2. Changes in quantitative HBsAg level form baseline through week 96 among the HIV/HBV-coinfected participants with or those without HBeAg positivity. The asterisk*indicates that the median level is significantly different from baseline ($p<0.05$).

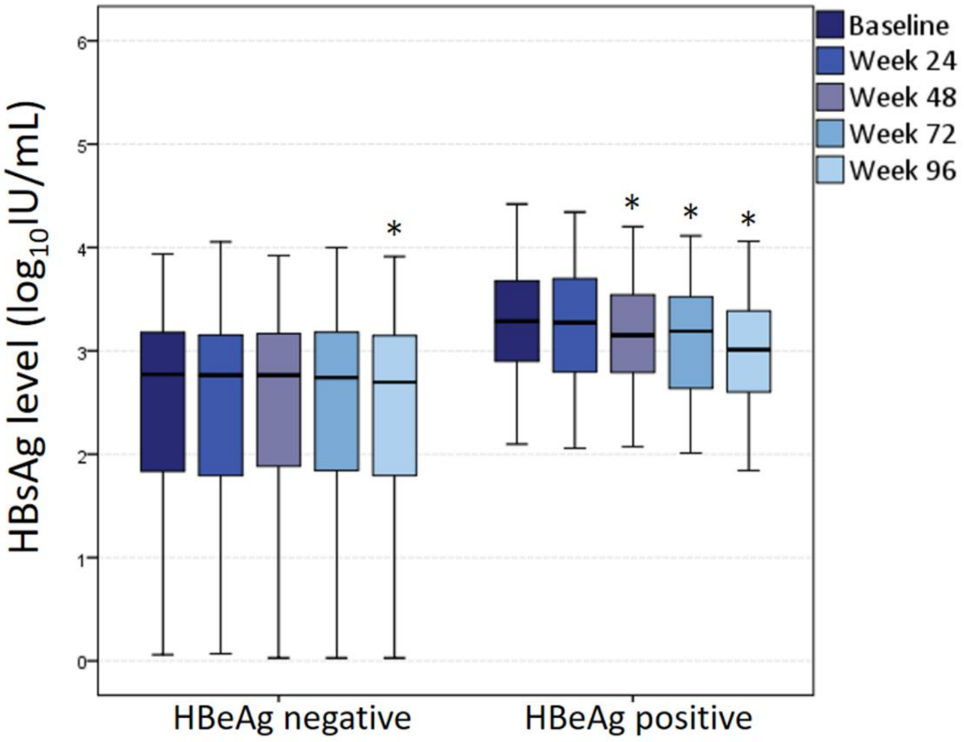

Supplementary Figure 3. Evolution of eGFR levels at week 96 of TAF-containing ART by different baseline eGFR (mL/min/1.73m^2) categories.

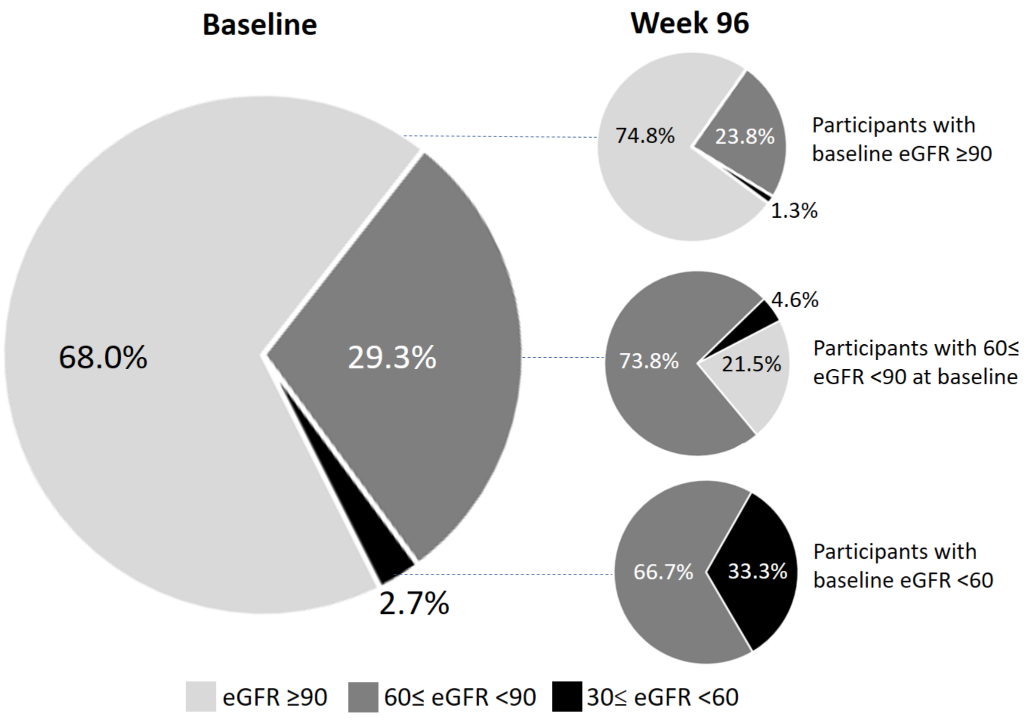

Supplement: Supplemental file 1 — Tables S1 to S5 and Fig. S1 to S3. Download spectrum.05125-22-s0001.pdf, PDF file, 1.1 MB [file spectrum.05125-22-s0001.pdf]
